# Clean and Safe Healthcare Environment: Knowledge, Attitude, and Practice of Infection Prevention and Control among Health Workforce at North Showa Zone Oromiya Region

**DOI:** 10.1155/2020/6021870

**Published:** 2020-10-30

**Authors:** Kemal Jemal, Ketema Gashaw, Tadele Kinati, Worku Bedada, Belete Getahun

**Affiliations:** ^1^Salale University, College of Health Sciences, Department of Nursing, Fitche, Ethiopia; ^2^Salale University, College of Health Sciences, Department of Public Health, Fitche, Ethiopia; ^3^Adama Hospital Medical College, Adama, Ethiopia; ^4^Canadian Physicians for Aid and Relief, Addis Ababa, Ethiopia

## Abstract

**Background:**

Infection prevention and control practice (IPCP) is essential for healthcare safety and quality service delivery. The Ethiopian government has already put in place programs and initiatives for clean and safe healthcare facilities. However, in the North Showa Zone of the Oromiya Region, the infection prevention and control practice level was not well understood. Therefore, this study aimed to assess the knowledge, attitude, and practice of infection prevention and control practice among the health workforce (HWF) in North Shoa healthcare facilities (NSHCFs) environment.

**Methods:**

Healthcare facility-based cross-sectional study design was employed. Structured and pretested self-administered questionnaires were distributed for 373 health workforce. Three hospitals and six health centers were randomly selected, and the study participants were selected by systematic sampling technique. Data were entered into Epi-data version 3.5.2 and then exported to SPSS version 23 for analysis. Multivariable logistic regression was performed to determine the associated factors with infection prevention practice, and a *p* value of less than 0.05 was considered statistically significant.

**Results:**

A total of 361 (96.8%) health workforce responded to self-administered questionnaires. About 55.70% of study participants had good knowledge, 59.3% of them had a positive attitude, and 46.8% had a good infection prevention practice. Age category of 20–29(AOR = 4.08, 95%, CI = (1.97, 8.49)), female participants (AOR = 3.87, 95%, CI = (1.91, 7.86)), single participants (AOR = 3.89, 95%, CI = (1.92, 7.87)), having greater than ten years of working experience (AOR = 3.10, 95% CI = (1.19, 8.10)), positive attitude (AOR = 10.07, 95% CI = (4.82, 21.05)), and availability of water at working area (AOR = 2.27, 95% CI = (1.18, 4.35)) were significantly associated with good infection prevention practice.

**Conclusion:**

In this study, a significant number of health workers had low knowledge, negative attitudes, and poor infection prevention practices. Female participants, higher work experience, a positive attitude, and water availability in the healthcare facilities were positively associated with infection prevention and control practice. Healthcare facilities should be continued capacitating the health workforce on infection prevention and control measures and equipping health facilities with infection prevention materials.

## 1. Introduction

Healthcare-associated infections (HCAIs) are a serious problem in healthcare settings, common causes of morbidity and mortality among the health workforce. In healthcare settings, around 1.4 million people are affected globally due to a lack of clean and safe healthcare facilities, which is 2 to 20 times higher in low-resource countries [1–5]. Healthcare facility-acquired infections occur in both high- and low-income countries due to poor healthcare cleanliness. In the USA, 1.7 million hospital-acquired infections contributed to 99,000 deaths each year, with annual costs of 25.0 to 31.5 billion dollars [6–8].

A study done in Nigeria found that 90% of healthcare workers (HCWs) were knowledgeable, 92.3% had a good attitude toward infection prevention, and 50.8% had good infection prevention and control practice [9]. A study done in Kenya indicated that only 17.8% of the study participants had adequate knowledge of the basic elements of infection prevention standard precautions [10].

In Ethiopia, a study done in Bahir Dar city indicated that 84.5% of the study participants were knowledgeable, 55.6% had a positive attitude, and 54.2% had good infection prevention practices [11]. Similarly, a study done in Addis Ababa among healthcare workers revealed that 55.4%, 83.3%, and 66.1% of the study participants had good knowledge, positive attitude, and good infection prevention practices, respectively [12]. Another study from Wolaita Sodo found that 99.3% of healthcare workers had good knowledge of infection prevention, 93.4% had a positive attitude toward infection prevention and control measures, and 60.5% of healthcare workers had good infection prevention and control practice [13].

Healthcare-associated infections can affect patients, patient families, healthcare workers, and supportive staff [14–16]. Accordingly, it can cause needless pain and suffering, long-term disability, excess death, prolong hospital admissions, and costly financial burden on the healthcare system [17–20]. Healthcare facilities' cleanliness is a significant predictor of the quality of healthcare and patient safety. Therefore, ensuring the facilities are comfortable and safe for patients, attendants, and staff [21, 22], in which healthcare providers are responsible for the healthcare facilities, creates an infection-free environment [23–25].

The Ethiopian government has taken initiatives that demand clean and safe healthcare facilities, standard precautions, and biohazard-free facilities [26]. However, most healthcare facilities are not clean; the image of an odorous, poorly organized institution with a filthy environment is common, particularly in rural healthcare facilities. There is not a clean and safe healthcare implementation due to a lack of supportive supervision. To date, there are limited continuous assessments concerning the safety and cleanliness of healthcare facilities in the North Showa Zone, Oromiya region. Therefore, this study aimed to determine the level of infection prevention and control practice and identify associated factors among the HWF in NSHCFs environment.

## 2. Methods

### 2.1. Study Design, Area, and Period

A facility-based cross-sectional study was applied from January to February 2019 in North Showa Zone, Oromiya Region. Fiche is the zonal town and located at 112 km from Addis Ababa in the North direction. The zone has two general hospitals, two district hospitals, 63 health centers, 268 health posts, seven medium clinics, 54 lower clinics, one drug store, 25 drug vendors, and three rural drug vendors.

### 2.2. Study Population

Health workforces who have been working for more than six months in North Showa Zone healthcare facilities were included in this study. We collected data from the health workforce (nurses, physicians, midwifery, medical laboratory professionals, health officers, pharmacists, ophthalmologists, and psychiatry nurses). Students who were on clinical attachment for less than six months and the health workforce on annual leave during data collection were excluded from the study.

### 2.3. Sample Size and Sampling Technique

The total sample size for this study was 373 as estimated using Epi-info version 7 power of 80% with a 95% CI and a 5% margin of error, taking a proportion of 36.3% from the study done in West Arsi district [27], and considering a 5% of nonresponse rate. From 67 healthcare facilities, three hospitals and six health centers were selected randomly. The total sample size was allocated proportionately to each facility based on the size of their health workforce. Again the allocated number, in turn, is proportionally assigned to each department. Eligible participants within the departments were selected by systematic sampling technique.

### 2.4. Data Collection Tools and Procedure

Data were collected using structured and pretested self-administered questionnaires. Two trained data collectors (diploma nurses) and a supervisor (bachelors) were involved in data collection. The questionnaires were adapted from the World Health Organization Infection Prevention and Control Assessment Framework tool and Ethiopian national guideline for the Clean and Safe Health Facilities Audit Tool for infection prevention and control practice components [28, 29]. The questionnaire contains the sociodemographic factors, infection prevention practice, knowledge, and attitude toward infection prevention.

Knowledge and attitude toward infection prevention and control practices were measured using ten questions which are “yes or no” for knowledge and agreed or disagreed for attitude; each correct answer “yes” or “agree” scored “1” and “no” or “disagree” scored “0” point for knowledge and attitude, respectively.

The outcomes of knowledge and attitude cut-off points were scored of eight and above (≥80%). A score greater than 80% was considered good knowledge and a positive attitude, and that less than 80% was considered poor knowledge and a negative attitude toward infection prevention and control practices [30, 31].

Infection prevention and control practice was measured using twelve questions (yes/no). Each correct answer scored five-point, and if not, zero. The outcome was calculated using the mean score as a cut-off point. A score greater than the mean was considered good infection prevention and control practice, otherwise, poor practice [28].

### 2.5. Data Processing and Analysis

Data were coded, edited, and entered into Epi-data version 3.5.2 and transported to SPSS version 23 for further analyses. The descriptive data analysis was done and presented as frequency, summary statistics, graph, and table. Bivariate and multiple variable logistic regressions were performed to identify associated factors. Both crude and adjusted odds ratios with 95% confidence interval were computed, and statistical significance was declared at *p* value ≤0.05.

### 2.6. Ethical Consideration

Ethical clearance was obtained from the Salale University Ethical Review Committee. Written permission was obtained from the North Showa zonal health bureau and each healthcare facility. Written informed consent was obtained from each study participant. To ensure the confidentiality of respondents, their names were excluded from the questionnaire.

## 3. Results

In this study, a total of 361 respondents were included, with a 96.8% response rate. More than three-fourths of the study participants have participated from the hospitals. Respondents' age range was from 20 to 42 years old, with a mean age of 29.07 (± standard deviation = 3.96). The higher proportion of 231 (64%) was in the age range of 20 to 29. Most study participants were male (66.8%), married (63.3%), BSc/MD (70.7%), and nurses (43.5%) that participated from hospitals. On the other hand, the salaries of the majority of participants in the health centers were between 1,080 and 4,999 Ethiopian Birr and had less than five years of experience (Table 1).

Two-thirds (238 (65.9%)) of the respondents were aware of safe injection guidelines, and only 30.8% and 26.1% had taken the training on safe injection from health centers and hospitals, respectively. More than one-third (134 (37.1%)) of the respondents had received training on handwashing practices, 34 (43.6) from health centers, and 100 (35.3) from hospitals. Three-seventh (42.7%) of the respondents had reported the availability of color-coded liner bags for waste segregation in their working environment. More than half (55.1%) and 69.8% of the respondents mentioned that a dust bin and adequate safety box were accessible in their working area, respectively. Merely, 47.4% of respondents had reported that health facilities had an active infection prevention and control committee (Table 2).

### 3.1. Knowledge of the Health Workforce to Infection Prevention and Control Practice

**Figure 1:**
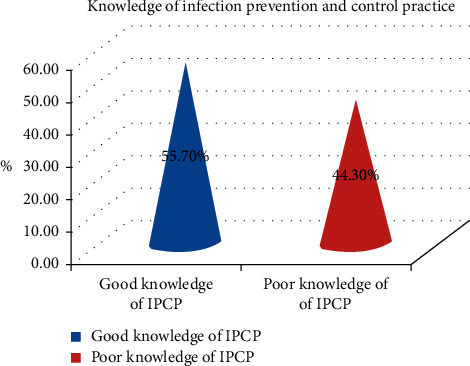
Knowledge of infection prevention and control practices among the HWF in NSHCFs environment, Oromiya region, Ethiopia, from January to February 2019 (*n* = 361).

The overall knowledge of health workforce for infection prevention and control practice was 55.7% (210) with 95% CI = (51.0 to 60.9) that had reported good knowledge of infection prevention and control practice, and 160 (44.3%) of the respondents had reported poor knowledge of infection prevention and control practice (Figure 1).

More than three-fourths of the study participants were aware of the availability of infection prevention and control policies and guidelines for healthcare workers. About 327 (90.6%) of the health workforce had known personal protective equipment to minimize healthcare-associated infections. More than half of the study participants had known that nosocomial infections could be transmitted through blood and body fluid contamination (54.6%) and precautions of safe disposal for needle syringes and any sharp wastes (57.3%). Two-thirds of the study participants knew the procedure of hand washing correctly (Table 3).

### 3.2. Attitude toward Infection Prevention and Control Practice among the Health Workforce

**Figure 2:**
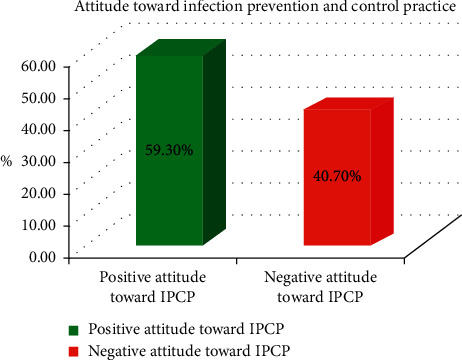
Attitude toward infection prevention and control practices among the HWF in NSHCFs environment, Oromiya region, Ethiopia, from January to February 2019 (*n* = 361).

The majority of the study participants had reported a positive attitude toward infection prevention, and control practice was 59.3% (214) with 95% CI = (53.7 to 64.3). In contrast, two-fifths of the health workforce had reported a negative attitude toward infection prevention and control practices (Figure 2).

Most of the study participants agreed that following standard operation procedures decreases the risk of contamination (70.1%), using personal protective equipment decreases HCAIs (73.4%), and using biohazard material is better for waste management (69.5%). More than three-fifths of the study participants agreed that recapping needle is the cause for needle prick injury in the healthcare facilities. The majority (287 (79.5%)) of the health workforce agreed to maintain ventilation in the ward or room by opening windows and doors to decrease infection transitions (Table 4).

### 3.3. Infection Prevention and Control Practice among the Health Workforce

**Figure 3:**
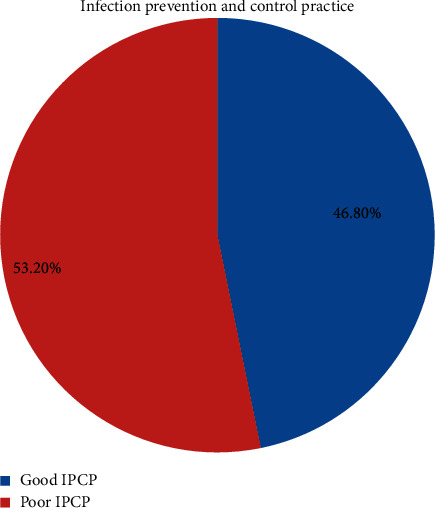
The prevalence of infection prevention and control practices among the HWF in NSHCFs environment, Oromiya region, Ethiopia, from January to February 2019 (*n* = 361).

Overall, less than half (169 (46.8%) with 95% CI = (41.8 to 52.1)) of the health workforce had reported good infection prevention and control practices, whereas 192 (53.2%) of the health workforce had reported poor infection prevention and control practices (Figure 3).

### 3.4. Factors Associated with Infection Prevention Practices

In the bivariable logistic regression analysis, the age category of 20 to 29, single participants, educational status of BSC/MD and MSC/specialty, female participants, nurse professionals, midwives and medical laboratory professions, good knowledge, and positive attitude were significantly associated with good infection prevention practice. Similarly, accessibility of dust bin, training on safe injection, and availability of water source in the working area were variables significantly associated with good infection prevention practice; whereas having good knowledge, accessible dustbins, and having training on injection safety were adjusted in multivariable logistic regression (Table 5).

In the multiple variables logistic regression analysis, the age range of 20 to 29 was four times more likely to practice infection prevention than older age [AOR = 4.08, 95%, CI = (1.97, 8.49)]. Female participants were four times more likely to practice infection prevention than male participants [AOR = 3.87, 95%, CI = (1.91, 7.86)]. The odds of being single were four times more likely to practice infection prevention and control than being married [AOR = 3.89, 95%, CI = (1.92, 7.87)]. Nurses, midwives, and medical laboratory professionals were increased by 2%, 4%, and 5% of infection prevention and control practice than health officers and other healthcare professionals. Those who have greater than or equal to ten years of work experience were three times more likely to practice infection prevention and control than those with less than five years of work experience [AOR = 3.10, 95% CI = (1.19,8.10)]. The study participants who have a positive attitude toward infection prevention and control practice were ten times more likely than a negative attitude [AOR = 10.07, 95% CI = (4.82,21.05)]. There was also a significant association between the availability of water in the work area and infection prevention and control practices [AOR = 2.27, 95% CI = (1.18, 4.35)] (Table 5).

## 4. Discussion

We set out to assess infection prevention and control practices among the healthcare facility workforce to better understand the possible area for improving infection prevention and control practice. We found that 169 (46.8%) of the respondents had reported good infection prevention and control practice. This result is in line with the study done in Mekelle, which found that 42.9% of the study participants had reported good infection prevention and control practice [32]. This finding is higher than that of the study conducted in the West Arsi district, which reported that 36.3% of healthcare workers had good infection prevention and control practices [27]. On the other hand, this result is lower than the results of studies in Addis Ababa health facilities (66.1%) and Debre Markos Referral Hospital (57.3%) [12, 33]. The difference might be related to the level and location of these facilities.

In our study, 55.7% of the respondents had reported good knowledge of infection prevention and control practice, which is similar to the studies conducted in Addis Ababa (55.4%) and West Arsi (53.7%) [27, 34]. This finding is higher than that of the study done in Amhara Regional State Referral Hospitals (40.7%) [35]. Conversely, this study is low when compared with the results of studies reported in Bahir Dar city (84.5%) [36] and Dessie Referral Hospital (95.19%) [37]. The difference might be attributed to training opportunities.

We found that the positive attitude toward infection prevention and control practice was 59.3%, which is in line with the study done in Bahir Dar city health institutions found that 55.6% of healthcare workers had reported a good attitude toward infection prevention and control practice [36]. This finding is higher than the study finding from Zabol Teaching Hospital, which revealed that 33% of HCWs had reported a good attitude toward infection prevention and control practice [38]. On the other hand, this finding is lower than that of the study conducted in Wolaita Sodo (93.4%) [13]. This difference may be due to variation in awareness of healthcare workers and study settings.

Our study found greater than half (52.6%) of study participants confirmed to be deficient in an active infection control committee in health facilities. Effective implementation of an infection prevention committee with the standardized guideline policy that emphasizes standard precautions' rational use may help check overtime infection prevention and control practice [39].

Providing on-job training and equipping the skill gap through training and knowledge transfer among healthcare providers and on-time assessment are the effective methods of practicing a safe and clean healthcare environment [40]. We found that only 27.1%, 37.1%, and 23.5% of the health workforce had received training on safe injection, hand washing, and waste segregation, respectively. A WHO evidence-based study recommended that trained healthcare providers significantly reduce HCAIs and improve their behavior and perception of infection prevention and control practice [41]. It is also enhancing the healthcare providers' knowledge, effective use of prepared guidelines, and safety precaution to save their staff, patients, and visitor in the healthcare environment [42].

In this study, we found a significant difference in infection prevention among different health facilities that affected the practices; females were more likely than males to practice infection prevention. This finding is similar to the studies done in Mekelle, Gondar, and Wolaita Sodo [13, 32, 43]. A study documented that females had willing to follow infection prevention and control guidelines, safety measures, and a desire to deliver good patient care [44].

On the other hand, young participants had a good infection prevention practice compared with those old ones. It is also similar to a study conducted in Mekelle [32], contradicting results reported in Debre Markos, where older ages are more likely to practice infection prevention [33]. This difference might be due to the age category cut point differences or the strength of study design.

The current study revealed a significant statistical association between being a nurse, midwife, and a medical laboratory. This finding is similar to the studies done in Addis Ababa and Mekelle where medical laboratory sciences and nurses were significantly associated with infection prevention and control practice [32, 34]. Nursing, midwifery, and medical laboratory professionals have a higher susceptibility to HCAIs as their working environment is more prone to infection. As a result, they use precaution measures and follow infection prevention and control guidelines, proper use of personal protective equipment, and accurate waste management.

There is also a significant statistical association in the health workforce with greater than ten years of work experience. This result is similar to the studies done in Bahir Dar city [36] and Debre Markos [33]. This may be explained due to increasing work experience; the healthcare providers learn from their previous errors experienced to adhere to infection prevention and control guidelines that may be beneficial for preventing and controlling HCAIs.

Education is a method of equipping the health workforce with up-to-date knowledge and skill of infection prevention and control practice with confidence utilization of recommended guidelines and the available supply [33]. As the educational level was increased, we found that infection prevention and control practices were better than the health workforce, which has a low educational level. This finding is similar to the study result in Debre Markos [33] but contradicts findings from a study done in the Amhara region [35]. The difference might be due to the self-reporting questionnaire and difference in the study area.

Our study also indicated that those who had a positive attitude toward infection prevention and control practices were significantly associated with good infection prevention and control practices. This finding is supported by a study reported from Addis Ababa [34]. The individual positive attitudes and beliefs may reflect healthcare workers' perception of the value of infection prevention and control guidelines in protecting them, their families, and their patients [45].

In this study, we found that 66.6% of the health centers and 69.3% of the hospitals had no running water in the healthcare facilities. However, only 31.3% of healthcare facilities have adequate running waters, which are statistically significant associated with infection prevention and control practices. This indicates that having running water in healthcare facilities is vital for infection prevention and control practices, which is useful for hand hygiene, equipment disinfected, and quality healthcare delivery. A previous study indicates that water provision in the healthcare setting is indispensable for clean procedure and survival of patient life; it plays an essential role in protecting human health from healthcare-associated infections [46].

## 5. Limitation of the study

This study has limitations; first, it does not show a causal relationship due to its cross-sectional nature. Second, the study has been conducted at healthcare facilities located only in the North Showa Zone healthcare facilities, the Oromiya region. Hence, our findings cannot be generalizable to the other regions of Ethiopia.

## 6. Conclusions

In this study, many health workers had low knowledge, negative attitudes, and poor infection prevention practices. Female participants, higher work experience, a positive attitude, and water availability in the healthcare facilities were positively associated with infection prevention and control practice. Effective performance is required to deliver safe and clean healthcare facilities for optimal outcomes, for both patients and healthcare providers. Healthcare facilities should be continued capacitating the health workforce on infection prevention and control measures and equipping health facilities with infection prevention materials. The Ethiopian Federal Ministry of Health should make an effort to promote clean and safe clinical practice following guidelines, optimizing the healthcare environment to ensure a working system that supports the effective implementation of infection prevention and control practices.

## Figures and Tables

**Table 1:** Sociodemographic and economic characteristics among the HWF in NSHCFs environment, Oromiya region, Ethiopia, from January to February 2019 (*n* = 361).

Variables	No. (%)		
	Total	Healthcare facilities	
		Health center	Hospitals
Overall	361 (100)	78 (21.6)	283 (78.4)
Age			
20–29	231 (64)	42 (53.8)	189 (66.8)
30–42	130 (36)	36 (46.2)	94 (33.2)
Sex			
Male	244 (67.6)	55 (70.5)	189 (66.8)
Female	117 (32.4)	23 (29.5)	94 (33.2)
Marital status			
Single	131 (36.3)	27 (34.6)	104 (36.7)
Married	230 (63.7)	51 (65.4)	179 (63.3)
Educational status			
Diploma	83 (23)	21 (26.9)	62 (21.9)
BSc/MD	249 (69)	49 (62.8)	200 (70.7)
MSc/specialty	29 (8)	8 (10.3)	21 (7.4)
Profession			
Nurses	159 (44)	36 (46.2)	123 (43.5)
Physicians	55 (15.2)	0	55 (19.4)
Midwives	43 (11.9)	11 (14.1)	32 (11.3)
MLS	59 (16.3)	14 (17.9)	45 (15.9)
Health officer	27 (7.5)	12 (15.4)	15 (5.3)
Others*∗*	18 (5.1)	5 (6.4)	13 (4.6)
Monthly salary			
1,080 to 4,999	229 (63.4)	46 (59.0)	183 (64.7)
5,000 to 10,058	132 (36.6)	32 (41.0)	100 (35.3)
Year of experience			
<5	210 (58.2)	38 (48.7)	172 (60.8)
5–9	107 (29.6)	29 (37.2)	78 (27.6)
≥10	44 (12.2)	11 (14.1)	33 (11.6)

*∗*Pharmacy, ophthalmologic nurse, and psychiatry nurse.

**Table 2:** Variables related to infection prevention and control practice among the HWF in NSHCFs environment, Oromiya region, Ethiopia, from January to February 2019 (*n* = 361).

Variables	No. (%)		
	Total	Healthcare facilities	
		Health center	Hospital
Overall	361 (100)	78 (21.6)	283 (78.4)
Aware safe injection guidelines			
Yes	238 (65.9)	57 (73.1)	181 (64.0)
No	123 (34.1)	21 (26.9)	102 (36.0)
Receives training on safe injection			
Yes	98 (27.1)	24 (30.8)	74 (26.1)
No	263 (72.9)	54 (69.2)	209 (73.9)
Aware handwashing guidelines			
Yes	243 (67.3)	54 (69.2)	189 (66.8)
No	118 (32.7)	24 (30.8)	94 (33.2)
Receive training on handwashing			
Yes	134 (37.1)	34 (43.6)	100 (35.3)
No	227 (62.9)	44 (56.4)	183 (64.7)
Running water in working order			
Yes	113 (31.3)	26 (33.3)	87 (30.7)
No	248 (68.7)	52 (66.6)	196 (69.3)
Training on waste segregation			
Yes	85 (23.5)	16 (20.5)	214 (75.6)
No	276 (76.5)	62 (79.5)	69 (24.4)
Color-coded liner bags in HCFs			
Yes	154 (42.7)	35 (44.9)	119 (42.0)
No	207 (57.3)	43 (55.1)	164 (58.0)
Dust bins accessible in HCFs			
Yes	199 (55.1)	49 (62.8)	150 (53.0)
No	162 (44.9)	29 (37.2)	133 (47.0)
Adequate safety box in HCFs			
Yes	252 (69.8)	51 (65.4)	201 (71.0)
No	109 (30.2)	27 (34.6)	82 (29.0)
Health facilities have an active infection control committee			
Yes	171 (47.4)	40 (51.3)	131 (46.3)
No	190 (52.6)	38 (48.7)	152 (53.7)

**Table 3:** Knowledge of HWF about infection prevention and control practice in NSHCFs environment, Oromiya region, Ethiopia, from January to February 2019 (*n* = 361).

Variables	Frequency	Percentage
Are you aware of the manual listing of the infection prevention and control policies and guidelines for healthcare workers?		
Yes	285	78.9
No	76	21.1
Do you know disinfection prevents hospital-acquired infection?		
Yes	305	84.5
No	56	15.5
Do you know antiseptic and sterilization techniques prevent hospital-acquired infection?		
Yes	290	80.3
No	71	19.7
Do you know personal protective equipment (mask, glove, and so on) minimizes HCAIs?		
Yes	327	90.6
No	34	9.4
Do you know the proper handling of working equipment decreases the risk of contamination?		
Yes	309	85.6
No	52	14.4
Do you know the precautions of safe disposal for needle syringes and any sharp wastes?		
Yes	207	57.3
No	154	42.7
Do you know the procedure of hand washing correctly?		
Yes	237	65.7
No	124	34.3
Do you know the effectiveness of handwashing in preventing HCAIs?		
Yes	260	72.0
No	101	28.0
Do you know when to perform handwashing?		
Yes	240	66.5
No	121	33.5
Do you know nosocomial infections can be transmitted through blood and body fluid contamination?		
Yes	197	54.6
No	164	45.4

**Table 4:** Attitude toward infection prevention and control practice among the HWF in NSHCFs environment, Oromiya region, Ethiopia, from January to February 2019 (*n* = 361).

Variables	Frequency	Percentage
Do you believe that following standard operation procedures decreases the risk of contamination?		
Agree	253	70.1
Disagree	108	29.9
Do you think ventilating the ward by opening windows and doors decreases infection transitions?		
Agree	287	79.5
Disagree	74	20.5
Do you think using personal protective equipment (PPE) decreases HCAIs?		
Agree	265	73.4
Disagree	96	26.6
Do you believe washing hands before and after contact with patients is important?		
Agree	286	79.2
Disagree	75	20.8
Do you agree that hospital facilities can be the source of infection in the absence of universal precaution?		
Agree	260	72.0
Disagree	101	28.0
Do you think separating needle and other types of waste is visible?		
Agree	279	77.3
Disagree	82	22.7
Do you think using biohazard material better for waste management?		
Agree	251	69.5
Disagree	110	30.5
Do you think that a patient's awareness about the transmission of microorganisms decreases the risk of HCAIs?		
Agree	248	68.7
Disagree	113	31.3
Do you agree that recapping is the cause of needle prick injury?		
Agree	227	62.9
Disagree	134	37.1
Do you believe that nosocomial infection can pose a serious outcome?		
Agree	231	64.0
Disagree	130	36.0

**Table 5:** Factors (crude and adjusted odds ratios and confidence intervals) associated with good infection prevention and control practice among the HWF in NSHCFs environment, Oromiya region, Ethiopia, from January to February 2019 (*n* = 361).

Variables	Infection prevention and control practice Poor	Infection prevention and control practice Good	Crude OR (95% CI)	*p* value	Adjusted OR (95% CI)	*p* value
Sex						
Male	145	99	1		1	
Female	47	70	2.20 [1.39, 3.42]	0.001^*∗∗*^	3.87 [1.91, 7.86]	0.001^*∗∗*^

Age						
20–29	108	123	2.08 [1.33, 3.24]	0.001^*∗∗*^	4.08 [1.97, 8.49]	0.001^*∗∗*^
30–42	84	46	1		1	

Marital status						
Single	59	72	1.7 [1.08, 2.58]	0.020^*∗*^	3.89 [1.92, 7.87]	0.001^*∗∗*^
Married	133	97	1		1	

Profession						
Nurse	99	60	0.22 (0.11, 0.46]	0.001^*∗∗*^	0.02 [0.01, 0.07]	0.001^*∗∗*^
Physician	21	34	0.59 [0.25, 1.39]	0.225	0.43 [0.14, 1.35]	0.147
Midwives	19	24	0.46 [0.19, 0.40]	0.048^*∗*^	0.04 [0.11, 0.15]	0.001^*∗∗*^
Medical laboratory science	41	18	0.16 [0.07, 0.38]	0.001^*∗∗*^	0.05 [0.02, 0.17]	0.001^*∗∗*^
Health officers and others	12	33	1		1	

Year of experiences						
<5	118	92	1		1	
5–9	53	54	1.31 [0.82 ,2.08]	0.261	0.90 [0.43, 1.87]	0.776
≥10	21	23	1.41 [1.73, 3.69]	0.043^*∗*^	3.10 [1.19, 8.10]	0.021^*∗*^

Educational status						
Diploma	30	53	1		1	
BSc/MD	141	108	0.43 [0.26, 0.72]	0.001^*∗∗*^	0.13 [0.55, 0.30]	0.001^*∗∗*^
MSc	21	8	0.22 [0.09, 0.55]	0.001^*∗∗*^	0.02 [0.01, 0.10]	0.001^*∗∗*^

Knowledge						
Poor	101	59	1		1	
Good	91	110	2.07 [1.35, 3.16]	0.001^*∗∗*^	1.10 [0.56, 2.17]	0.775

Attitude						
Negative	106	41	1		1	
Positive	86	128	3.85 [2.45, 6.05]	0.001^*∗∗*^	10.07 [4.82, 21.05]	0.001^*∗∗*^

Aware HW guidelines						
No	68	50	1		1	
Yes	124	119	1.31 [0.84, 2.03]	0.239	1.46 [0.78, 2.74]	0.243

Water in the working area						
No	147	101	1		1	
Yes	45	68	2.20 [1.40, 3.46]	0.001^*∗∗*^	2.27 [1.18, 4.35]	0.014^*∗*^

Dustbins accessible						
No	97	65	1		1	
Yes	95	104	1.63 [1.07, 2.48]	0.022^*∗*^	1.44 [0.76, 2.66]	0.250

Training injection safety						
No	152	111	1		1	
Yes	40	58	1.98 [1.24, 3.18]	0.004^*∗*^	1.96 [0.90, 4.25]	0.090

^*∗*^Significant association (*p* value <0.05), ^*∗∗*^*p* value ≤0.001. HW: hand washing.

## Data Availability

The datasets used and/or analyzed during the current study are available from the corresponding author upon reasonable request.

## Abbreviations

CASH: Clean and safe health facility
HCFs: Healthcare facilities
HCWs: Healthcare workers
HWF: Health workforce
IPCP: Infection prevention control practice
NSHCFs: North Shoa healthcare facilities
SPSS: Statistical package for social sciences
WHO: World Health Organization.
